# Enhancing missense variant pathogenicity prediction with protein language models using VariPred

**DOI:** 10.1038/s41598-024-51489-7

**Published:** 2024-04-07

**Authors:** Weining Lin, Jude Wells, Zeyuan Wang, Christine Orengo, Andrew C. R. Martin

**Affiliations:** 1 Division of Biosciences, Institute of Structural and Molecular Biology, University College London, London, UK; 2 Department of Computer Science, University College London, London, UK; 3 College of Computer Science and Technology, Zhejiang University, Zhejiang, China

**Keywords:** Computational models, Machine learning, Protein analysis, Proteome informatics

## Abstract

Computational approaches for predicting the pathogenicity of genetic variants have advanced in recent years. These methods enable researchers to determine the possible clinical impact of rare and novel variants. Historically these prediction methods used hand-crafted features based on structural, evolutionary, or physiochemical properties of the variant. In this study we propose a novel framework that leverages the power of pre-trained protein language models to predict variant pathogenicity. We show that our approach VariPred (Variant impact Predictor) outperforms current state-of-the-art methods by using an end-to-end model that only requires the protein sequence as input. Using one of the best-performing protein language models (ESM-1b), we establish a robust classifier that requires no calculation of structural features or multiple sequence alignments. We compare the performance of VariPred with other representative models including 3Cnet, Polyphen-2, REVEL, MetaLR, FATHMM and ESM variant. VariPred performs as well as, or in most cases better than these other predictors using six variant impact prediction benchmarks despite requiring only sequence data and no pre-processing of the data.

## Introduction

A large portion of genetic variation is represented by single nucleotide variants (SNVs). SNVs occur in both protein coding and non-coding regions, while protein-coding SNVs can be further divided into synonymous and non-synonymous (nsSNVs) types. Synonymous SNVs do not change the amino acid sequence of the resulting protein while non-synonymous SNVs (nsSNVs) do.

Missense mutations, in which a single amino acid is replaced by another, are the most common type of nsSNV (the others leading to truncation or extension). There is a long history of using physicochemical and evolutionary information to predict whether a given missense mutation is disease-causing^1–3^. Nonetheless it remains a major challenge to predict pathogenicity.

To tackle these challenges, many computational tools based on supervised machine learning techniques have been developed to predict the potential impact of variants. These compute deleterious scores based on dozens of biological properties of variants, such as evolutionary conservation^3–5^, biochemical properties of amino acids^6,7^, and structural features of proteins^8,9^.

However, typically only a subset of variants can be annotated with all the features. This is especially true for tools that require a protein structure. There are 200 million protein sequences in the UniProt databank dated 12th Oct 2022 (see: https://www.ebi.ac.uk/uniprot/TrEMBLstats), but only 200,000 experimentally-determined protein 3D structures stored in the Protein Data Bank (see: https://research.rutgers.edu/news/new-collaboration-between-rcsb-protein-data-bank-and-amazon-web-services-provides-expanded). This indicates that only approximately one in a thousand proteins have a reliable, experimentally resolved structure. For example, a commonly used predictor, Missense3D, can only structurally annotate 1965 and 2134 variants out of 26,884 disease-associated and 563,099 neutral variants, using structures from the PDB^9^. Even given the increase in structural coverage using predicted protein structures from AlphaFold2^10^, the accuracy of AlphaFold2 in predicting the structure of proteins with shallow multiple sequence alignments (MSAs) or orphan proteins, is questionable^11^ and structure-based predictors may need to be re-trained for different levels of predicted quality obtained from AlphaFold2.

Traditional computational variant effect predictors, such as PolyPhen-2 (Polymorphism Phenotyping v2)^6^ are some of the most popular tools for clinical researchers. PolyPhen-2 was trained on protein sequence alignments and exploits known or predicted protein three-dimensional structural information to predict the impact of nsSNPs on protein structure and function.

FATHMM (Functional Analysis Through Hidden Markov Models)^12^ took a somewhat different approach to previous predictors being trained using Hidden Markov Models, with a range of features including conservation and physicochemical properties, to predict the impact of genetic variations on protein function. It is capable of handling a wide range of variations, including those in non-coding regions and is therefore valuable to clinical researchers.

In addition to the aforementioned techniques, ensemble methods have also been developed for predicting the pathogenicity of missense variants. REVEL (The Rare Exome Variant Ensemble Learner) and MetaLR, which uses Logistic Regression (LR), stand out as two representative ensemble models that were considered the best in the Critical Assessment of Genome Interpretation (CAGI) competitions^13^. REVEL, an ensemble method aimed at predicting the pathogenicity of missense variants, combines scores from 13 distinct pathogenicity prediction tools. MetaLR was developed by integrating scores from 10 different tools.

In the latest genome interpretation assessment competition (CAGI-6), a novel predictor named 3Cnet was ranked top in the SickKids clinical genomes and transcriptomes panel (see: https://www.3billion.io/blog/3billion-wins-in-cagi6-a-global-artificial-intelligence-genome-interpretation-contest/). 3Cnet^14^ is a deep artificial neural network Long Short-Term Memory (LSTM)-based model, which utilises multiple protein features including MSAs, amino acid physicochemical properties, and other features such as motifs and active sites as the input^14^. As PolyPhen-2, FATHMM, REVEL, MetaLR and 3Cnet are all trained for predicting the clinical significance of missense variants, and 3Cnet has reported state-of-the-art performance in this field, we selected these as state-of-the-art methods against which to benchmark our approach.

Most recent novel protein data-representation approaches take inspiration from language models that have yielded ground-breaking improvements in natural language processing (NLP). In particular, the Transformer neural network architecture^15^, can learn contextualised word representations from a large amount of unlabelled text data and has achieved state-of-the-art performance for several NLP tasks. In the life sciences, most protein language models (pLMs) use Transformer architectures which were developed for NLP, but were subsequently trained on protein sequences with the goal of deciphering the ‘natural language’ of proteins.

pLMs such as ProtT5^16^, ESM-1b^17^, ESM-1v^18^ and ESM-2^19^ have been trained on a large corpus of protein sequences with the objective of predicting masked amino acids given the context of the non-masked sequence. These pLMs are pre-trained using a masked language modelling objective. During the pre-training, 15% of residues were randomly masked out from the input sequences, and the model predicts which amino acid type is most likely to be present at each masked position.

This results in a learned feature representation called an ‘embedding’ for each residue position in the protein sequence. The embeddings of these sequence-based pre-trained models have been shown to encode useful bio-physical information, such as residue conservation^20^ amino acid hydrophobicity, protein structure class^16^ and protein functional properties^21^.

Even though these models were pre-trained without using evolutionary information, it has been shown that the methods achieve a similar performance to MSA-based models for various tasks while also reducing the computational cost.

**Figure 1:**
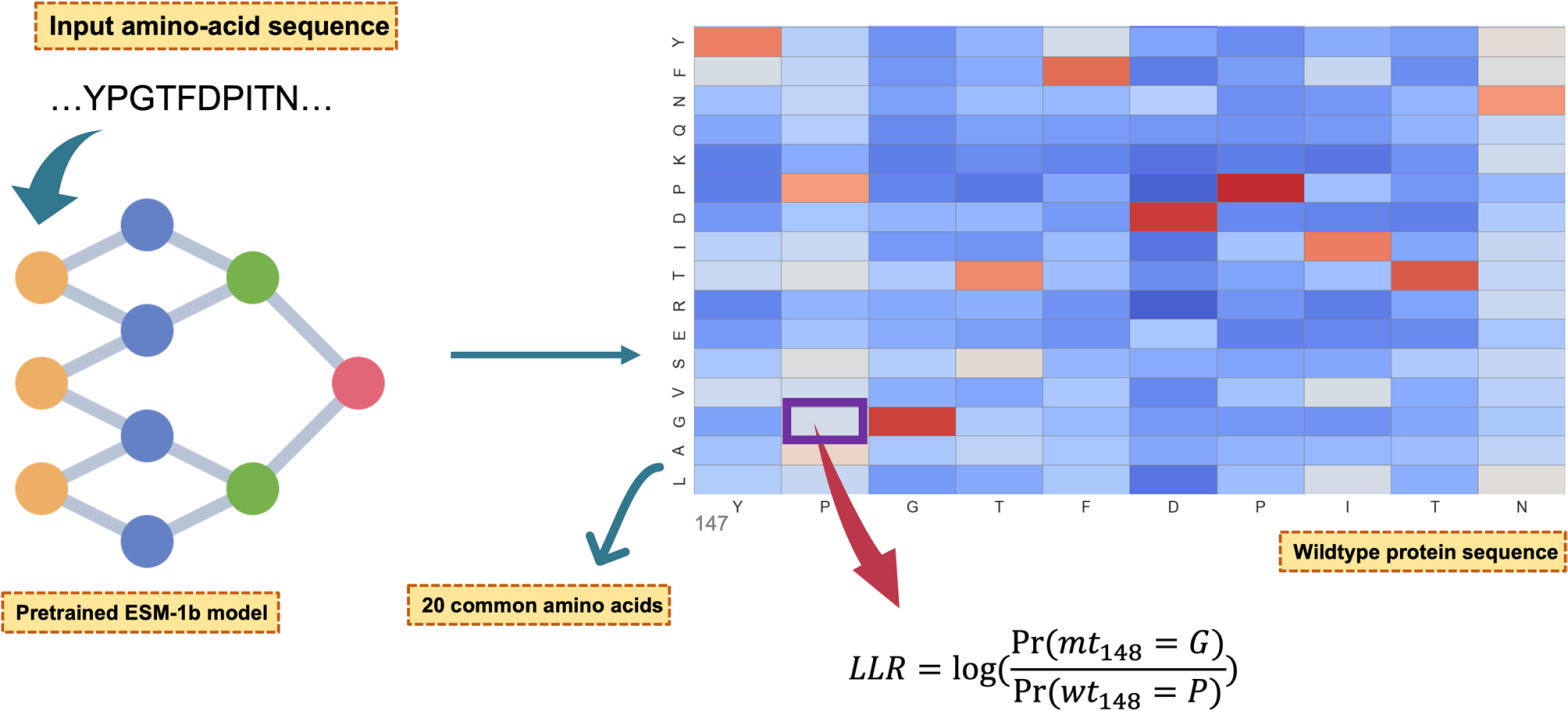
Illustration of how to calculate the log-likelihood ratio (LLR) from ESM models. The input for the pLM is an amino-acid sequence while the output is the log-likelihood ratio calculated based on the probabilities of the wildtype amino acid and the mutant amino acid occurring at the given position. Note, the heatmap showing in this figure was artificially generated for demonstration purposes. Pr, Probability; wt_148_ = P, probability that the residue occurring at the 148th position in the input wildtype (wt) protein sequence is a Proline (P); mt_148_ = G, probability that the 148th residue was mutant type (mt) Glycine (G).

Recent studies have used experimental data to evaluate the performance of pLMs in predicting the functional effects of variants^17,20,22,23^. However, to date, only one study (‘ESM variant’) has used a pLM to predict the clinical significance of a mutation^22^. ‘ESM variant’ uses the ESM-1b pre-trained pLM without requiring any supervised training. Given that ESM-1b was trained to predict the likelihood of each amino-acid type at each position, it is possible to use these likelihoods as a proxy for how well tolerated an amino-acid change is likely to be at the mutation site. ‘ESM variant’ constructs a pathogenicity score for a given mutation by using the ESM-1b likelihoods for the wildtype and mutant type amino acids at the mutated position (Fig. 1).

This study extends the research on pLMs by proposing a novel methodology and architecture. We conduct a comparative analysis of alternative pLM models including the recent ESM-1v and ESM-2 models which were trained on larger protein datasets. We compare performance of our model against two traditional machine learning-based models including Polyphen-2 and FATHMM, and two novel deep learning based-methods ‘ESM variant’ and 3Cnet using the ClinVar dataset. One downside of supervised machine learning models is that they can be prone to overfitting such that they can perform poorly with new sequences not seen during the training stage, and where the training data contains genes for which all variants have the same class label (benign/pathogenic). A previous study identified this as the ‘Type 2 data circularity’ problem^24^. We investigate whether the predictors are prone to bias from Type 2 data circularity using two additional benchmarks: SwissvarFilteredMix and VaribenchSelectedPure.

Our model, VariPred, uses a novel twin-network pLM architecture combined with a trained classification module to achieve the highest classification performance on two variant classification benchmarks. The twin neural network framework (sometimes called a Siamese network) describes an approach where two comparable inputs are each passed through the same network. In our case, we pass the mutant and wildtype sequences through the pLM to generate embeddings for each residue position. Subsequently, we concatenate the two embeddings for the wildtype and mutant type residues that occur at the mutation position. These paired embeddings are used as input features to a lightweight feed-forward classification module which is trained on the labelled data. As a result of the transformer network architecture and the pLM’s pre-training objective, contextual information from the entire sequence is incorporated into the per-residue embedding. At the same time, selecting only the embeddings for the residues at the mutation position allows the classification module to focus on information which is specific to the mutation site.

## Results

### Comparing the performance of LLR, embeddings and LLR + embeddings

The ‘ESM variant’ method^22^ only uses the Log-likelihood ratio (LLR) feature derived from ESM-1b to predict the clinical significance of missense variants. Here we evaluate the performance for this task, using LLRs derived from two other pLMs: ESM-1v and ESM-2.

**Figure 2:**
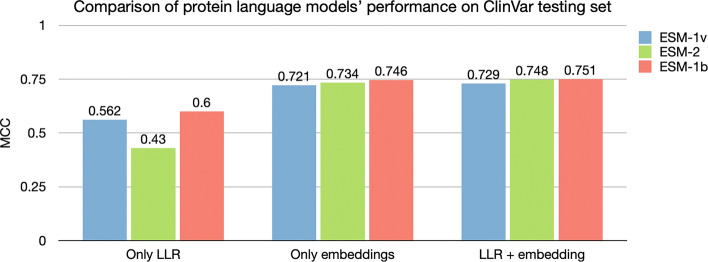
Comparison between models under different testing situations. Comparison of the LLR and embedding features for three protein language models. The baseline is using ‘Only LLR’ to predict pathogenicity of variants; For ‘Only embeddings’ we used amino acid embeddings as input to a shallow Feed-forward neural network (FNN) to predict the pathogenicity of variants; ‘LLR + embeddings’ concatenates the LLR feature as the last column of the amino acid embedding matrix, and then performs variant classification by using this extended matrix as input to the FNN. LLR: Log-likelihood ratio.

We first tested models using the ClinVar test set. For pLMs which only use the LLR for prediction, ‘ESM variant’ (using ESM-1b) has the best predictive performance, with a Matthews correlation coefficient (MCC) score of 0.600, followed by ESM-1v (0.562) and ESM-2 (0.430) (Fig. 2).

In addition to using the LLR threshold to differentiate pathogenic variants from benign, our VariPred predictor uses a shallow Feed-forward Neural Network (FNN) trained on the pLM embeddings for the wildtype and mutant sequence. We observe that this approach significantly improves the performance of all protein language models. ESM1b remains the best performing model, with an MCC score of 0.746, followed by ESM-2 (0.734) and ESM1v (0.721) (Fig. 2).

When we combine the LLR together with the embeddings, the performance of all models improved further. ESM-1b still has the best performance with an MCC score of 0.751, ESM-2 scored 0.748, and ESM-1v achieved 0.729 (Fig. 2). Therefore, in the following experiments to compare the performance with other tools, we chose ESM-1b as the feature extractor for our model, VariPred. We trained our model with both embeddings and LLRs as input.

### Comparison between models using the ClinVar test set

To evaluate VariPred’s performance on clinical data, we compared the performance of VariPred with other tools on the ClinVar test set.

**Figure 3:**
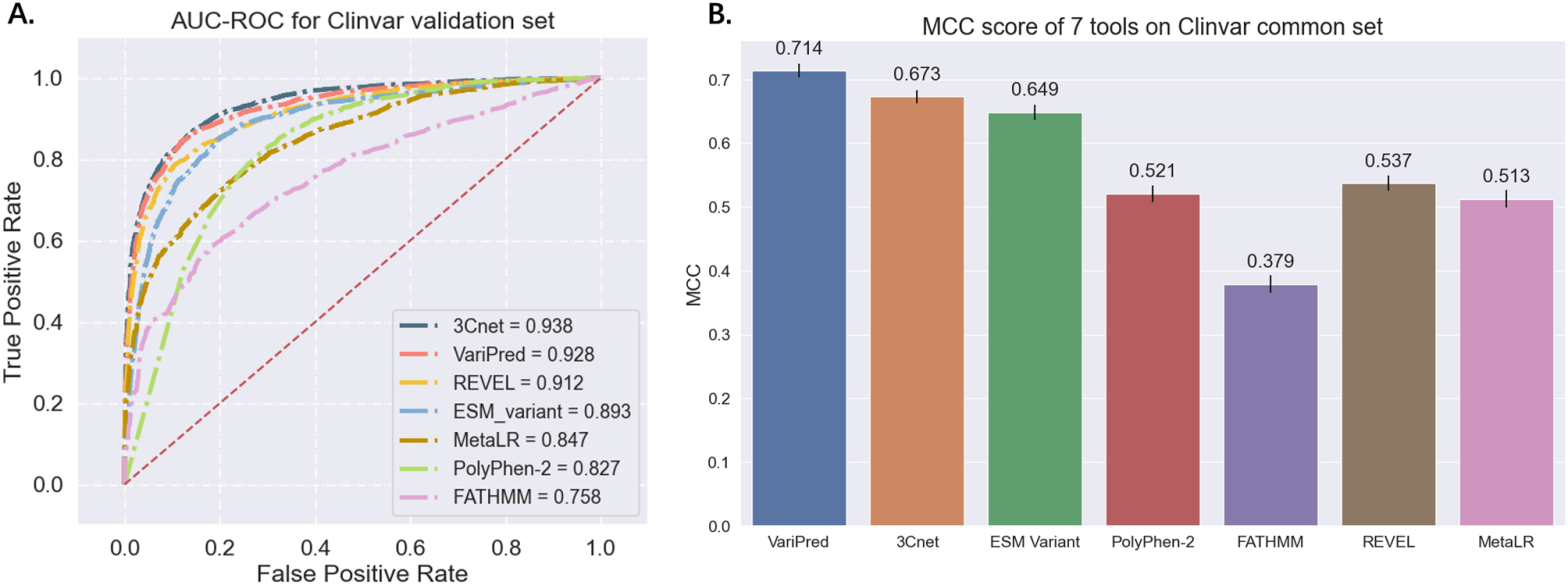
Comparing the performance of pathogenicity predictors using the ClinVar validation set. (**A**) AUC-ROC curve plot for the seven predictors. (**B**) MCC score for the predictors being tested in this study. The error bars depict the 95% confidence interval for the MCC score, as computed from 10,000 bootstrap sampling iterations.

Comparing against other methods, VariPred has the best performance with an MCC of 0.714. 3Cnet is closest to matching the performance of VariPred with an MCC of 0.673, followed by ‘ESM variant’ (MCC = 0.649), two ensemble predictors REVEL and Meta-LR2 with MCC scores of 0.537 and 0.513, respectively, and PolyPhen-2 (MCC = 0.521), while FATHMM has the lowest performance with an MCC of 0.379 (Fig. 3). In addition, VariPred only requires protein sequence information as input, while 3Cnet requires multiple features including MSAs, amino acid physicochemical properties, and protein features such as motifs or active sites.

We observed that on the ClinVar dataset, 3Cnet's AUC-ROC is higher than that of VariPred, while its MCC is lower. This discrepancy might stem from the inherent differences in how these two metrics evaluate model performance. AUC-ROC is calculated as the area under the curve plotted with True Positive Rate (TPR) and False Positive Rate (FPR) at various thresholds. However, AUC-ROC does not consider the shape of the curve or the threshold used for classification. Rather it focuses on the model's overall performance across all possible thresholds. Consequently, for imbalanced datasets, the ROC curve may provide an overly optimistic performance estimate. In contrast, MCC considers all four quadrants of the confusion matrix at a single threshold (as would be used in an actual predictor), making it a balanced metric even in cases of class distribution imbalance.

However, another reason could be that the default threshold for 3Cnet was not optimal for these datasets. We therefore assessed the effect of adjusting the 3Cnet threshold to improve performance and observed that when this was done there was no significant difference in the MCC values between VariPred and 3Cnet (with 3Cnet obtaining a slightly higher performance, see Supplementary).

In addition, to test whether the model’s performance is artificially inflated by simply learning and predicting the majority class label for certain genes we created an additional ‘balanced label’ subset of the ClinVar validation set (see “Methods”: “Additional ‘balanced-label’ test set”) based on the ClinVar validation set, ensuring an equal number of positive and negative variants for all genes. Results on this subset showed that both models had similar AUC-ROC (0.88), while VariPred has a much higher MCC (0.623) compared with that of 3CNet (0.567).

#### Adjusting for gene overlap in training and test sets

The common standard in the existing literature on supervised models for variant impact prediction is to allow the same gene/protein to occur in the training and test sets as long as there is no overlap in the variant. This is the approach taken in the 3Cnet paper. The justification is that a clinically useful model should be able to leverage information from homologous and identical proteins to infer the pathogenicity of previously unseen variants. To investigate whether VariPred's prediction accuracy is inflated due to protein homology, we retrained the VariPred ESM-1b model on a new split of the ClinVar data and evaluated the performance on a non-redundant test set (no sequence identity above 30% across the train and test datasets). We find that the MCC changes from 0.75 to 0.65 (ROC AUC 0.93 to 0.91) when changing from the original train-test split to the no-homology split. This gives a measure of the model’s expected performance when predicting on genes without any annotation in the training data. While we are unable to benchmark the other supervised models on the non-homology train/test split, as this would require retraining the other models, we note that VariPred’s performance on the no-homology split is still better than the best unsupervised method.

### Results for Type 2 data circularity test

We compared all predictors with two public benchmarks, the SwissvarFilteredMix and VaribenchSelectedPure test sets, to evaluate if any of the models are affected by the Type 2 data circularity problem.

**Figure 4:**
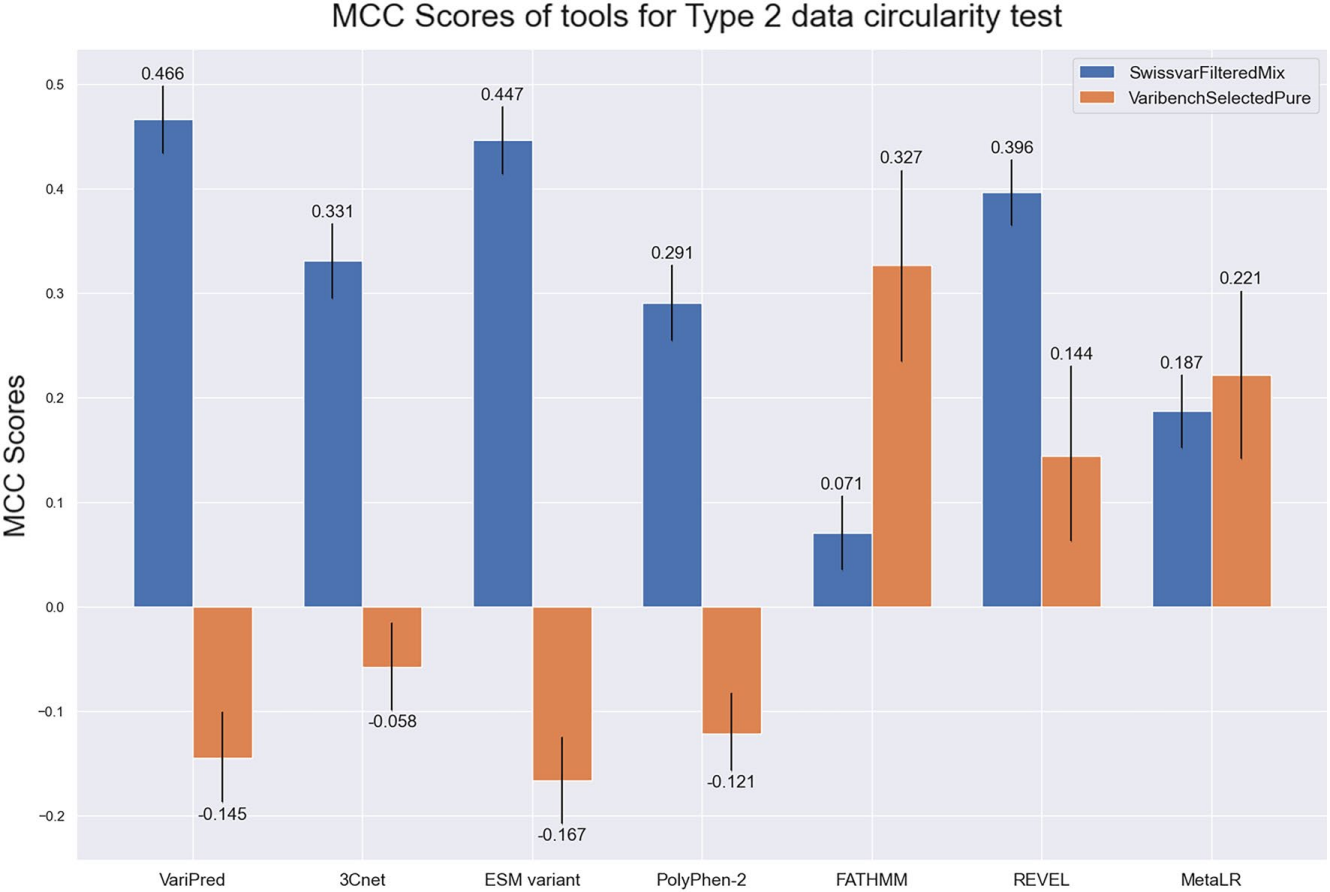
Type 2 data circularity problem test. Comparing the performance of pathogenicity predictors using the public test set, SwissvarFilteredMix and VaribenchSelectedPure. Predictors were all tested in the common datasets of both SwissvarFilteredMix and VaribenchSelectedPure. The error bars depict the 95% confidence interval for the MCC score, as computed from 10,000 bootstrap sampling iterations.

In this evaluation, FATHMM has the highest accuracy in VaribenchSelectedPure, while having a much higher performance in VaribenchSelectedPure (MCC = 0.327) than SwissvarFilteredMix set (MCC = 0.183), which is consistent with previous research^25^ (Fig. 4). In addition, MetaLR’s performance in VaribenchSelectedPure, for which the MCC score equals 0.221, is higher than SwissvarFilteredMix set (MCC = 0.187). This suggests that traditional machine learning-based methods, FATHMM and MetaLR, are both affected by the Type 2 data circularity problem where the model is learning features of the gene and ignoring the specifics of the variant (see “Methods”: “Additional ‘balanced-label’ test set” for a more detailed explanation). In contrast, our model, VariPred, has the highest performance in SwissvarFilteredMix (MCC = 0.466) (Fig. 4), indicating that VariPred is not confounded by the Type 2 data circularity problem.

While PolyPhen-2 demonstrates comparable performance to 3Cnet using the SwissvarFilteredMix set, it is crucial to note that Swiss-Prot forms part of the training data for PolyPhen-2. Consequently, the observed MCC score of 0.291 for PolyPhen-2 applied to SwissvarFilteredMix may be inflated due to train/test overlap.

Among the three deep-learning-based tools, all predictors demonstrated superior performance on SwissvarFilteredMix as compared to VaribenchSelectedPure. Notably, 3Cnet exhibits the lowest MCC score among them, registering a value of 0.331.

## Discussion

We tested three different pLMs (ESM-1b, ESM-1v, ESM-2) and showed that ESM-1b was the best predictor for pathogenicity of single-position missense variants (Fig. 2). Using the ClinVar test set, our VariPred predictor which combined LLR and residue embeddings generated by ESM-1b has the best performance achieving an MCC of 0.714 and AUC-ROC of 0.928 without using any additional biological features and not being confounded by Type 2 data circularity (Fig. 3).

In principle, since ESM-1v and ESM-2 were pre-trained using a much larger protein sequence dataset, they should have a broader view on the mutability landscape of proteins than ESM-1b. The ESM-1v and ESM-2 authors state that both ESM-1v and ESM-2 are sufficient to conduct the missense mutation pathogenicity prediction without any further training^18,23^. Nonetheless, we observed better performance using the ESM-1b model. We speculate that this may be a result of the ESM-1b pre-training dataset being more closely aligned with the relatively narrow set of (human only) proteins that are included in ClinVar.

Reports suggest that for predicting the functional (rather than clinical) effects of variants, which are in the form of a continuous scalar value, ESM-1v and ESM-2 have a better performance^18,23^. However, recent comments suggest that ESM-1b performs better in some other tasks, such as structure prediction^26^. In this study, we showed that ESM-1b outperforms two other state-of-the-art predictors in predicting the binary clinical significance of missense variants.

In the comparison between representative traditional machine learning-based methods (PolyPhen-2 and FATHMM), ensemble models incorporated with traditional machine learning methods (REVEL and MetaLR) and deep learning-based models (VariPred, 3Cnet and ‘ESM variant’) for the task of clinical variant impact detection, the accuracy of models based on deep learning models is far higher than that of models based on traditional machine learning methods (Fig. 3). The lowest MCC score in the deep learning model, ‘ESM variant’, is higher than the relatively high MCC score of REVEL in traditional machine learning methods by around 30%, while supervised deep learning methods (VariPred and 3Cnet) out-perform the unsupervised learning method (‘ESM variant’).

In the Type-2 data circularity test, we noticed that traditional machine learning based models are prone to the bias from the Type-2 data circularity problem. FATHMM, which was not trained on Swiss-Prot dataset, has the highest accuracy in VaribenchSelectedPure (MCC = 0.327) compared with the SwissvarFilteredMix set (MCC = 0.071) while the ensemble model MetaLR also demonstrates a higher performance in VaribenchSelectedPure than SwissvarFilteredMix set, of which the MCC scores are 0.221 and 0.187, respectively (Fig. 4). Meanwhile, none of the deep learning-based methods have a better performance in the VaribenchSelectedPure set. In particular, our model, VariPred, has the highest performance in SwissvarFilteredMix (MCC = 0.466) but a lower performance in VaribenchSelectedPure (MCC = − 0.145) (Fig. 4), indicating that VariPred is not confounded by the Type 2 data circularity problem. This suggests that, compared with deep learning-based methods, traditional machine learning based predictors are more easily affected by the Type 2 data circularity problem where the model is learning features of the gene and ignoring the specifics of the variant.

**Figure 5:**
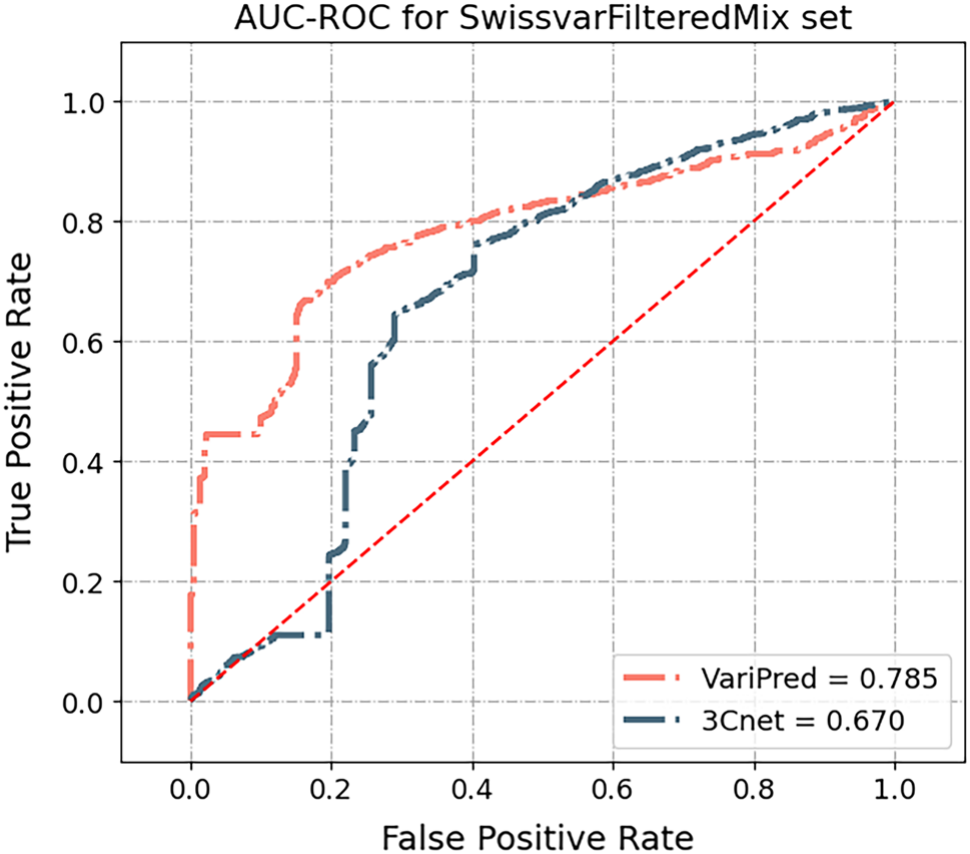
AUC-ROC curve plot for VariPred and 3Cnet on SwissvarFilteredMix dataset.

From the test results on the ClinVar ‘balanced label’ subset, we found that VariPred is less susceptible than 3Cnet to simply predicting the majority class label for genes that have been included in the training data. According to our evaluation on another balanced dataset, SwissvarFilteredMix, 3Cnet's performance on either MCC or AUC-ROC was significantly behind VariPred (Figs. 4, 5). Thus VariPred is the top performing method in two out of three test sets (ClinVar-class-balanced and SwissVar) and is competitive on a third test set (ClinVar) where we observe no statistically significant difference between VariPred and 3Cnet (Fig. 5**)**.

Preparing the input features for 3Cnet is a difficult task. 3Cnet relies on features based on 85 biophysical properties retrieved from the SNVBox database. However, not all variants can be mapped with features from SNVBox as it has not been updated since 2011, resulting in missing sequences and problems with changed RefSeq IDs. This may lead to uncertainty in the consistency between the retrieved feature and the data entry. Additionally, only the NP codes which are included in the provided transcript ID list can be transformed into a 3Cnet prediction dataset.

In comparison to other models, VariPred requires only the most fundamental information for each data entry: the wildtype protein sequence and mutation information including *which* residue is being mutated at *which position* in the wildtype sequence into *which* mutant amino acid. Without the need for further dataset preparation, such as MSA construction or feature retrieval, making predictions on a dataset of 5000 new variants take 30 min with a 12 GB GPU, such as the Nvidia GTX 1080Ti.

A key feature of VariPred, fine-tuned based on pLMs, is its ability to fit the pathogenicity data extremely well using only sequence data for training. When compared with 3Cnet, VariPred removes dependencies on MSAs and externally computed features, requiring only the protein sequence as input. Thus, while other prediction models directly exploit structural and/or evolutionary information, their performance in pathogenicity prediction was not as good as that of large language models relying solely on sequence information. Our VariPred results appear to validate previous research suggesting that pLMs pre-trained on a vast array of sequences have implicitly learned the structural^16^ and evolutionary information^20^ of proteins.

We note that performance on the SwissvarFilteredMix and VaribenchSelectedPure test sets was lower than performance on ClinVar for all models, which is consistent with previous studies^25^. Comparing the SwissVarFilteredMix dataset with the latest ClinVar dataset (2022-12) we found that 17% of entries have an inconsistent label (e.g. ‘uncertain’ in SwissVarFilteredMix versus ‘positive’ in ClinVar) and 81% do not have a label in the ClinVar dataset, whilst 2% of variants have the opposite label. Meanwhile, VaribenchSelectedPure also has label discrepancies when compared with ClinVar. Of the 69 variants shared between the ClinVar and VaribenchSelectedPure datasets, 22 have contradictory labels. The reason is not immediately clear, but both VaribenchSelectedPure and SwissVarFilteredMix are old datasets. As all models perform badly in these two datasets, we suspect that the labels of the same variants in these two tests may have been changed in ClinVar.

This could explain why the performance of all predictors drops in these two datasets, as the inconsistent data would reduce the evaluated prediction performance of the predictors. This could be a result of different criteria for labelling (e.g. how partial penetrance variants are classified) or labels changing owing to newly available evidence. We note that other authors have been critical of using the VariBenchSelectedPure dataset for benchmarking. For example, quoting from Wang and Wei^25^: “It is useful to note that the performance of the tools on VaribenchSelectedPure cannot be compared directly because this dataset was biased and the labels of variants in this dataset were at least partially artificial. It was only constructed for testing whether a prediction method was confounded by type 2 circularity.”^25^.

Inconclusive and contradictory pathogenicity labels are an argument in favour of unsupervised methods such as ‘ESM variant’. Even though we observed lower performance when compared with supervised methods such as VariPred and 3Cnet, it is important to note that the unsupervised methods are not prone to bias introduced by training dataset selection and labelling issues.

VariPred is specifically trained for predicting pathogenicity rather than functionality. This distinction is crucial, with pathogenicity being a more downstream concern, while functionality deals with more upstream mechanisms. Consequently, our training prioritises pathogenicity data over functionality datasets. Nevertheless, we postulate that our research can be complemented by in vitro functional protein assays, including deep mutational scanning experiments, which may provide additional insights into disease-causing variants. Additionally, our homology tests have demonstrated that when VariPred is retrained by training with sequence similarity level less than or equal to 30% compared to that in the test set, it still achieves remarkable performance on the test set (MCC = 0.645, AUC-ROC = 0.907). Therefore, we believe that a retrained VariPred can offer valuable insights about disease causing variants on a gene not previously encountered.

Several researchers have posited that variant predictors might yield enhanced accuracy for specific genes or diseases^25,27^. Thus, in the future, we will evaluate VariPred’s performance on specific genes associated with various diseases as well as differential pathogenicity prediction—i.e. predicting different pathogenic phenotypes caused by mutations in the same protein^27^.

Currently, VariPred only uses sequence information to predict pathogenicity. In future we will evaluate the effect of including structural information in the predictor. A similar strategy of predicting mutation effects using protein embeddings has been implemented for aiding engineering of enzymes by directed evolution and for aiding protein design^28,29^, but has not yet been explored in the prediction of the clinical significance of missense mutations. Incorporating both sequence and structural information is likely to improve VariPred’s ability to classify missense variants.

Applying a more biologically meaningful data augmentation strategy may add more diversity into the training set. Conservation information is one of the most powerful features for predicting protein stability and functional effects^30^. In the study of 3Cnet, the artificial pathogenic-like variants were generated simply by considering the amino acid frequency and the number of gaps. However, a good conservation scoring scheme depends on multiple components, of which the most important include amino acid frequency, residue similarity (biophysical properties), sequence similarity (considering sequence redundancy and MSA depth), the number of gaps in the MSA, and the concept of ‘compensated pathogenic mutations’ (CPDs), which refers to mutations occurring in different species that are tolerated because of compensating mutations^31^. Indeed, it may be possible to exploit the LLR output from ESM-1b to suggest compensatory mutations and use the final VariPred output to evaluate their effect. Therefore, in the future, it may be worth investigating whether such a combination of data augmentation and synthetic data strategies can further improve the performance of VariPred.

In summary, VariPred only requires the native and mutated sequence and, using protein language model encoding, is able to outperform state-of-the-art methods that use features including structural information and multiple sequence alignments.

## Methods

### Dataset preparation

To avoid having to retrain 3Cnet we opted to use the same train/test split as the 3Cnet authors. The only modifications that we made were to exclude the 3Cnet simulated data from our model’s training set and remove some variants from the test set which appear to have been inadvertently included by the 3Cnet authors in both the train and test sets.

#### Training set

The training dataset used in 3Cnet consists of three parts: (1) clinical data stored in the clinical database ClinVar, (2) common missense variants retrieved from the population database GnomAD, and (3) a set of simulated pathogenic data generated by the 3Cnet authors. The simulated data are based on amino acid conservation determined from MSAs, built using sequences from the RefSeq database. We chose to exclude the simulated data from our training dataset and work with the subset of the 3Cnet training data sourced from ClinVar and GnomAD.

The ClinVar dataset used by 3Cnet was downloaded from the ClinVar database via the FTP link (version 2020-4). In total, 72,470 curated missense variants were selected according to the criteria of known molecular consequences and reliable review status. Specifically, only variants with the GRCh37 assembly version and labelled with ‘missense variants’ were collected, and those with unreliable review status, containing strings with ‘no assertion’, ‘Conflicting’, ‘no interpretation’, and ‘Uncertain’ were all excluded. Data labelled with ‘pathogenic’ or ‘likely pathogenic’ were all considered as pathogenic variants. Similarly, variants with any submission reported as either ‘benign’ or ‘likely benign’ were defined as neutral. After filtering out low-quality data, 72,470 variants (22,337 pathogenic and 50,133 benign) remained.

The GnomAD dataset prepared by the 3Cnet group (file downloaded using FTP: gnomad.exomes.r2.0.2.sites.vcf.gz) consists of 60,614 exome-derived variants. These variants have a minor allele frequency (MAF) higher than 0.1% and each was filtered by requiring a ‘PASS’ annotation, which ensures the quality of the variant, i.e. high confidence genotypes. Since these variants are found in the genome of supposedly healthy people, they are typically regarded as benign variants^32^. However, even though the 3Cnet authors regard variants with a MAF ≥ 0.1% as neutral, we cannot exclude the possibility that some of these variants have undetected (or partial penetrance) pathogenic effects since a MAF of 1% is usually used in defining a ‘polymorphism’.

Each datapoint included in these three datasets was annotated with a specific RefSeq NP code (protein record identifier in the protein sequence database) and the mutant information in the HGVSp term by the 3Cnet group, e.g. NP_689699.2:p.Gly56Ser. For each RefSeq NP code, the corresponding curated wildtype protein sequence was also provided by the 3Cnet group. For each variant in the dataset, the input for the model consists of both wild-type and mutant sequences, target mutated position, wildtype amino acid and the mutant amino acid. The final output of the model is a binary label, where 0 indicates that the mutation is benign and 1 indicates pathogenic.

#### Test set

We noted that some variants (the same gene with the same mutation) were repeated between the 3Cnet train and test datasets. This problem arises from splitting the data based on variant information given in the HGVSp term. We found that some proteins annotated with different NP codes are in fact the same isoform, with the same wildtype protein sequence. To avoid having to retrain 3Cnet we chose to remove the 1767 duplicated variants from the ClinVar test set and a further 900 variants which were duplicated between the GnomAD training dataset and the ClinVar test set.

As a result of removing these duplicates, the processed training dataset consists of 72,466 variants from ClinVar and 59,018 variants from GnomAD. In total 17% of variants were labelled as pathogenic. The test set is comprised of data from ClinVar only and consists of 21,125 entries with 45% of variants labelled as pathogenic (Supplementary Table 1). Here, we ensure that the training and test sets do not have any variants which are the same.

When fetching pre-computed prediction results from FATHMM, PolyPhen-2, REVEL and MetaLR, we noted that some variants were not available. To ensure all methods were benchmarked with the same test set, we tested all predictors on the common test set, consisting of 12,853 variant datapoints in total.

#### Additional ‘balanced-label’ test set

In the comparative analysis of performance metrics, it is observed that the AUC-ROC score of 3Cnet surpasses that of VariPred, whereas the MCC score of 3Cnet is inferior. This divergence in performance metrics might be attributed to a specific characteristic of the dataset used in the full set. Notably, certain genes within this dataset exclusively comprise variants of a singular type, exclusively classified as either benign or pathogenic. This situation suggests that the predictive model may predominantly assimilate information at the gene level rather than the variant level. In essence, the model tends to predict all variants of a particular protein as either benign or pathogenic. This phenomenon aligns with what is known as a type 2 data circularity problem.

To test whether model performance was artificially inflated by learning to predict the majority class for genes with imbalanced class labels, an additional subset was constructed, termed the ‘balanced-label’ subset, derived from the Clinvar validation set. This subset was constructed to include only those genes that exhibit both classes of variants and a strict balance was enforced in the number of each label for these genes. To illustrate, genes that solely consisted of variants labelled as 0 or as 1 were excluded from the test set. In the genes that remained, an equilibrium was ensured by taking a random subsample from the majority class of the same size as the minority class.

#### Testing for Type 2 data circularity bias

Grimm et al*.*^24^ point out that effective benchmarking of clinical variant prediction can be confounded by circularity arising from overlap between the train and test sets. Type 1 data circularity arises when the same variant is included in the train and test set. Type 2 circularity arises from the same genes being included in the train and test set, even where the individual mutations are distinct. Type 2 data circularity bias is a particular problem where the data includes genes where labels are imbalanced towards one class. This scenario can give rise to predictors that ignore the specific details of the mutation merely recognising genes which are oversampled as pathogenic or benign in the training data. In order to assess the models’ propensity to overfit to genes in this way, we followed the approach of Grimm et al*.* and tested predictors using the SwissvarFilteredMix dataset and the VaribenchSelectedPure public benchmark. These datasets are used together to test whether performance is confounded by type 2 data circularity.

The SwissvarFilteredMix dataset consists of proteins with at least one type of label from each class. By contrast, in the VaribenchSelectedPure dataset, each protein only has one type of variant class, either all benign or all pathogenic. If a model learns to predict based on characteristics of the gene and ignores the specifics of the variant, then it will typically show inflated performance on the VaribenchSelectedPure dataset while showing low performance on SwissvarFilteredMix. It is important to emphasise that the performance of tools on VaribenchSelectedPure should not be directly compared, as this dataset has inherent biases and its variant labels are, to some extent, artificially generated. The primary purpose of creating this dataset was to test whether a prediction method could be confounded by type 2 circularity. The 3Cnet and VariPred training set includes 67% of the genes that were in the VaribenchSelectedPure test set, and therefore have the type 2 circularity that we are trying to test.

The VaribenchSelectedPure and SwissvarFilteredMix datasets contain information on chromosome number, base substitution position, reference nucleotide base, altered nucleotide base, Ensembl protein ID and the ground-truth label. Some sequences are not consistent with the mutation information, possibly because there has been a new isoform since the two benchmarks were generated in 2016. Therefore, we annotated NM codes (mRNA record identifiers in the Nucleotide database) for each variant using the latest version of the ANNOVAR software^33^, with the transcript-based annotation set for the RefSeq Gene (assembly version hg19; updated 2020-08-17 at UCSC). We then retrieved the corresponding protein isoform sequences (wildtype sequences) using the Entrez.efetch module included in Biopython (version 1.80) with Python 3.9. Using the original protein isoform sequences and the corresponding variant information, we generated the mutant sequences for each variant. This gave a SwissvarFilteredMix test set with 1153 benign variants and 1023 pathogenic variants, and a VaribenchSelectedPure set with 3629 benign variants and 2122 pathogenic variants.

There are problems with 3Cnet in generating features for some variants from these two benchmark datasets. To predict a variant’s pathogenicity with 3Cnet requires three components: the HGVSp term including the NP code and the mutation information, the NP code corresponding to the wildtype sequence, and 85 biological features retrieved from the SNVBox database^34^. However, this information is not recorded in the SNVBox database for some of the variants. Consequently, 3Cnet is not able to give a prediction for these variants. We therefore dropped these variants, leaving 1742 variants in the SwissvarFilteredMix and 5159 variants in VaribenchSelectedPure test sets for the evaluation of 3Cnet. We ensured that no variant was repeated between the train and test sets i.e. no type 1 circularity.

Moreover, owing to the absence of some human genes in both the precomputed datasets of PolyPhen-2 and FATHMM, we tested all tools using the common variants between SwissvarFilteredMix and VaribenchSelectedPure sets. Eventually, 1603 out of 2176 variants from SwissvarFilteredMix (865 benign vs. 738 pathogenic) and 1837 variants from the VaribenchSelectedPure set (1760 benign vs. 77 pathogenic) remained that could be tested across all predictors.

### Feature extraction and model setup

In order to identify the most suitable pLM for differentiating pathogenic variants from benign, we tested the most widely used pre-trained models including ESM-1b, ESM-1v and ESM-2. ESM-2 has several versions with different parameter sizes, ranging from 8 × 10^6^ to 15 × 10^9^. According to a previous study, the performance of the model does not increase with model size, and models with 650 × 10^6^ parameters appear to have the best ability to extract per-residue features^23^. Therefore, we chose ESM-2 with 650 × 10^6^ parameters for our analyses.

#### Extract embeddings by pLMs

ESM-1b and ESM-1v are BERT-style encoder-based Transformers, which limit the input length to 1022 amino acids. ESM-2 does not have this sequence length limitation, but using longer sequences is computationally prohibitive. Moreover, the Rotary Position Embedding strategy used in ESM-2 only considers the word embeddings and their neighbours, limiting any advantage of larger windows. Therefore, we designed a sequence truncation strategy which is consistent with such encoding methods and limits the maximum length to 1022 in all 3 models.

The official ESM tokenizer package pads the length of shorter sequences to 1022 internally, but transforms the length back to the true sequence length during data processing. For sequences longer than 1022, if the mutation is within 1022 residues of either the N-terminus or the C-terminus, 1022 residues counting from the end were retained; if the mutation index occurs more than 1022 residues from both termini, 510 neighbours from the N-terminal side and 511 from the C-terminal side of the mutated residue were selected, resulting in sequences having 1022 residues.

Transformer-based pLMs provide features in two forms: the probability of each amino acid type occurring at each position in the sequence, and a dense vector-embedded representation of each position in the sequence. Owing to the self-attention mechanism of the Transformer architecture, the embedding can incorporate contextual information from the entire 1022 residue window. We followed a similar approach to ESM-Variant, and for each mutated position we extracted the log likelihood ratio (LLR) and the embedded representation of the wild-type and mutant type residue.

The LLR was calculated using the ESM likelihood of the mutant and wildtype amino acid at the target position conditioned on the model receiving the wildtype sequence as input, using the formula shown in Fig. 1.

**Figure 6:**
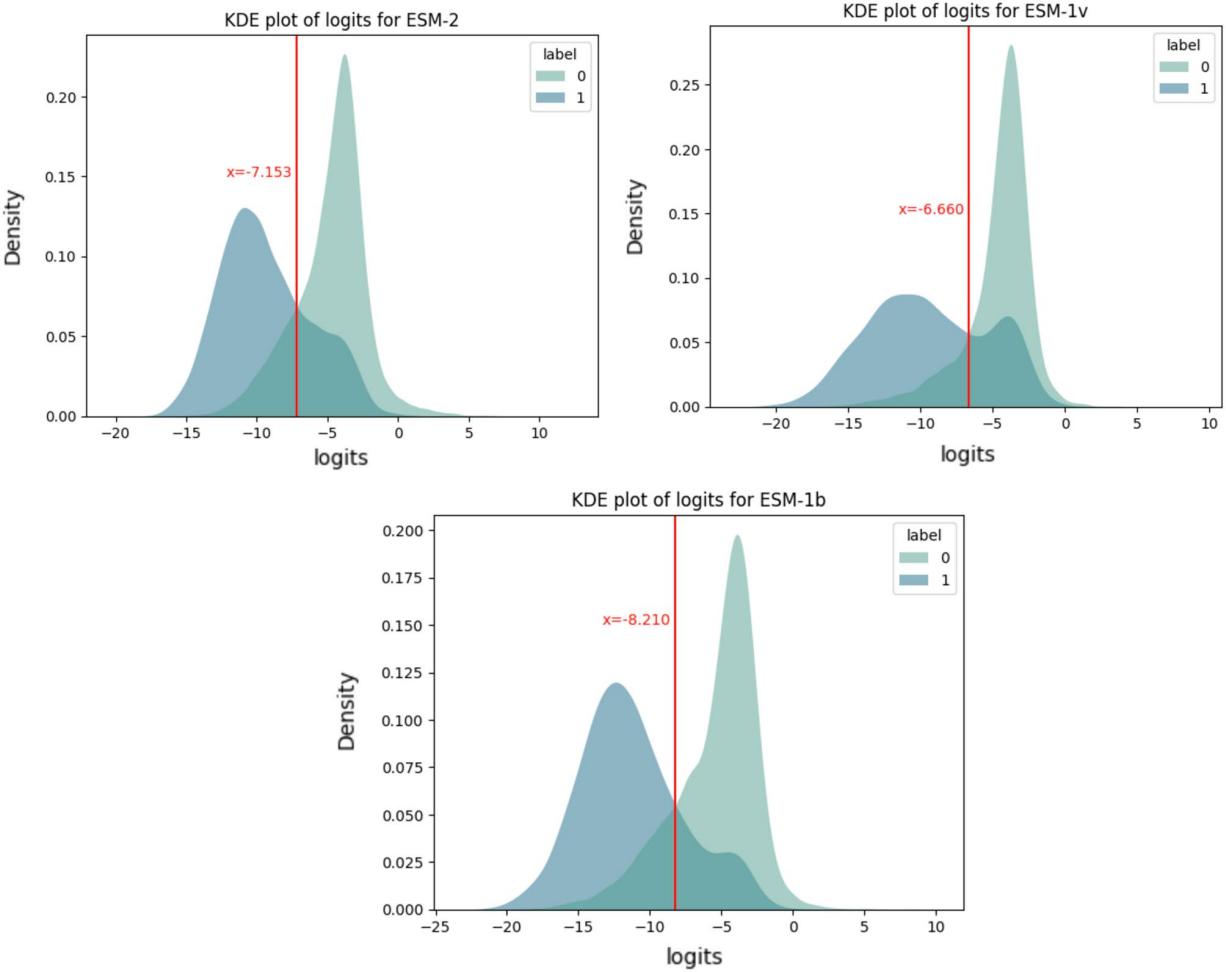
The intersections of the two LLR distributions among three models. The LLR values obtained from ESM-2, ESM-1v, and ESM-1b were plotted as a kernel density estimation (KDE) distribution along with their corresponding true values. This visualization enables the differentiation between pathogenic and benign variants.

Amino acids which frequently occur at a target position, typically have a comparatively high likelihood. Thus, if the mutant type’s likelihood is significantly lower than the wild type, this serves as an indicator that the mutation is problematic, while mutant residues with high likelihoods typically have similar physiochemical properties and are therefore less likely to affect protein stability or function. The ‘ESM variant’ method^22^, which uses the LLR generated by ESM-1b to discriminate variant pathogenicity, suggested that an LLR threshold of − 7.5 is sufficient to detect pathogenic variants. Considering the potential variability of 'cross-points' i.e. the overlap in distributions between two labels in the validation set, across different models and across different test sets, we optimised the performance of models via calculating the specific cross-points for the three models: ESM-2, ESM-1v, and ESM-1b, with respective values of − 7.513, − 6.660 and −8.210—on the training set utilised in our study (Fig. 6). The ESM model with the best performance will then serve as the feature extractor for VariPred. Each position in the sequence is represented by embeddings of a 1280-word dimension, extracted from the chosen ESM model.

**Figure 7:**
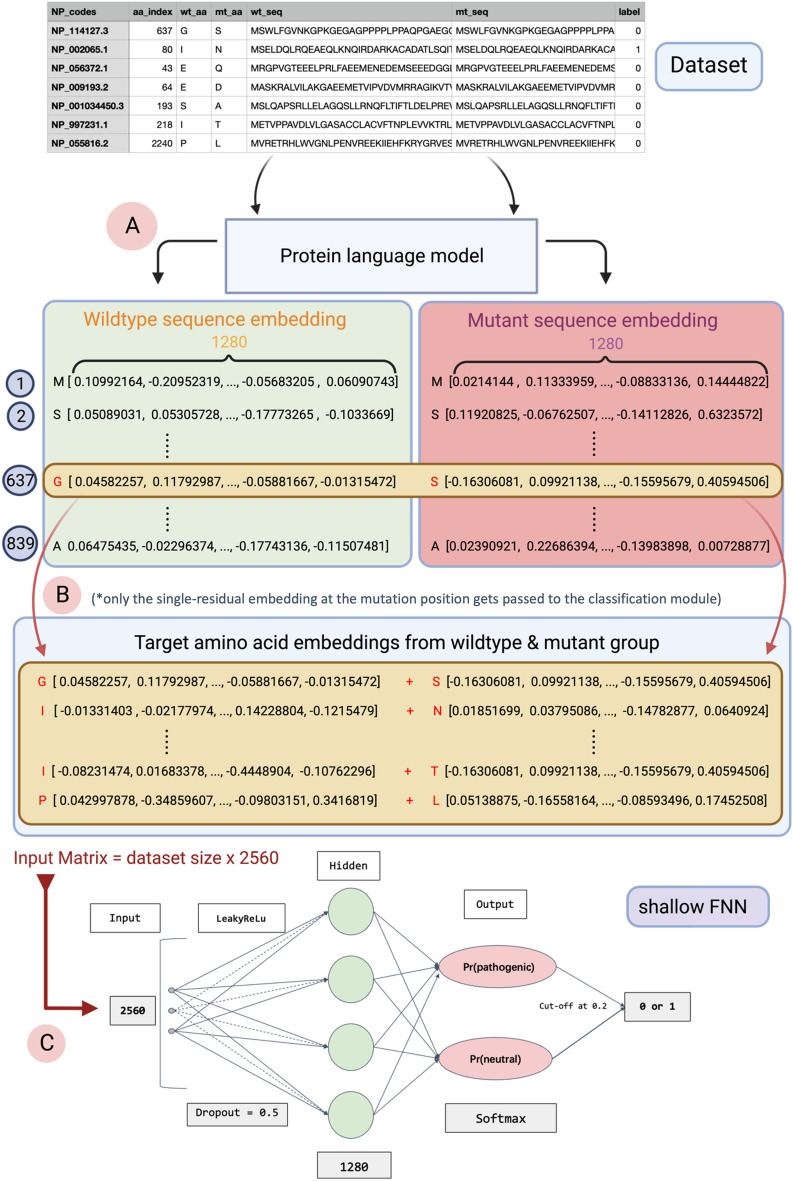
Schema of workflow for training VariPred. (**A**) In the first step, each wild-type protein sequence and the corresponding mutant protein sequence are fed into the pLM separately. The pLM generates a per-residue embedding for each amino acid. The output is the matrix of sequence embedding, with dimensions sequence length x embedding dimension. (**B**) Only the embeddings of the amino acids at the mutated position are used and joined giving an embedding dimension of 2560. The concatenated embeddings for each observation are combined to give an embedding matrix with dimensions dataset size ×2560. (**C**) The embedding matrix is fed as the input into a Feedforward Neural Network (FNN), and two probabilities are then output identifying if the given variant belongs to the pathogenic or benign group. Note that if the LLR feature is appended the input matrix is dataset size ×2561.

The schema of data processing and model generation is shown in Fig. 7. To obtain the embeddings of each sequence pair (wildtype and mutant protein sequences), all sequence pairs were fed into the pLM. For example, if a wildtype sequence consists of 100 amino acids, two embedding matrices (one for amino acids in the wildtype sequence, the other for amino acids in the mutant sequence) with dimensions 100 × 1280 would be generated (Fig. 7A).

We hypothesised that the embedding of the target amino acid at the mutation position would be the most informative. Therefore, we only took the embedding of the amino acid at the mutated position from both wildtype and mutant sequences. These two embeddings were then concatenated horizontally such that each data entry is represented as a vector with dimensions 1 × 2560. Feeding the training dataset (192,575 entries) into the ESM-1b pre-trained model will generate a wildtype-mutant concatenated amino acid embedding representation matrix with a size of (192,575 × 2560) (Fig. 7B). These embeddings are expected to capture fundamental biological features, related to protein function or structural stability.

To investigate whether combining LLR and embeddings would increase performance, we appended the LLR to the last column of the embedding matrix, which increased the dimension from 2560 to 2561 (Fig. 7C).

#### Feed-forward neural network

We created a classification module by including a shallow feed forward neural network (FNN) as the decoder/classifier for the pLM. This was trained on the class labels without updating parameters in the pLM. During the hyperparameter tuning process, we tried increasing the depth of the FNN as well as trying multiple sets of learning rates and drop-out rates. The final FNN, which gave optimal performance, consists of one hidden layer, a LeakyReLu activation function, and one output layer with the dropout rate set at 0.5 and learning rate set at 0.0001 (Fig. 7C). The input layer of the feed forward neural network has 2560 nodes (2561 if LLR is appended), while the hidden layer contains 1280 nodes, and the output consists of 2 nodes with a SoftMax layer to ensure the output probabilities sum to 1 for binary classification of benign/pathogenic. Only the pathogenic output node was considered and a value of 0.2 was selected as a threshold for predicting the pathogenic class. This threshold was selected by optimizing the MCC on the validation set during model training and the low value results from the high skewness of the dataset towards neutral variants.

### Evaluation metrics

Accuracy, Precision-Recall, F1-score, MCC (Matthews correlation coefficient) and AUC-ROC (area under curve of the receiver operating characteristic) are some of the most popular metrics for evaluating binary classifiers. Since MCC takes all outcomes (true and false positives and negatives) into account (Eq. 1), it is less sensitive to class imbalance and is also more informative about the classifier’s performance at a given threshold^35^. In contrast, other measures are more sensitive to imbalance^36^ and the AUC-ROC gives a view of the overall performance of a classifier (across a range of thresholds) rather than the actual performance in a classification problem.

1 $$MCC=\frac{TP\times TN-FP\times FN}{\sqrt{\left(TP+FP\right)\left(TP+FN\right)\left(TN+FP\right)\left(TN+FN\right)}}$$

Therefore, we applied MCC as the main metric to measure the performance of predictors studied in this research, while using AUC-ROC as an auxiliary indicator.

### Supplementary Information

Supplementary Information.

## Data Availability

All data and code required to reproduce the model and analysis in this study are available at https://github.com/wlin16/VariPred.git.
